# Intestinal S100/Calgranulin Expression in Cats with Chronic Inflammatory Enteropathy and Intestinal Lymphoma

**DOI:** 10.3390/ani12162044

**Published:** 2022-08-11

**Authors:** Denise S. Riggers, Corinne Gurtner, Martina Protschka, Denny Böttcher, Wolf von Bomhard, Gottfried Alber, Karsten Winter, Joerg M. Steiner, Romy M. Heilmann

**Affiliations:** 1 Small Animals Department, College of Veterinary Medicine, Leipzig University, 04103 Leipzig, Germany; 2 Institute of Animal Pathology, Department of Infectious Diseases and Pathobiology (DIP), Vetsuisse Faculty, University of Bern, 3012 Bern, Switzerland; 3 Institute of Immunology/Molecular Pathogenesis, Center for Biotechnology and Biomedicine, College of Veterinary Medicine, Leipzig University, 04103 Leipzig, Germany; 4 Institute of Veterinary Pathology, College of Veterinary Medicine, Leipzig University, 04103 Leipzig, Germany; 5 Veterinary Pathology Center, 80689 Munich, Germany; 6 Institute of Anatomy, Medical Faculty, Leipzig University, 04103 Leipzig, Germany; 7 Gastrointestinal Laboratory, College of Veterinary Medicine and Biomedical Sciences, Texas A&M University, College Station, TX 77843, USA

**Keywords:** calgranulins, calgranulin C, calprotectin, chronic enteropathy, chronic inflammatory enteropathy, feline, food-responsive enteropathy, intestinal lymphoma, small-cell lymphoma, S100A8/A9, S100A12

## Abstract

**Simple Summary:**

Intestinal diseases in cats are complicated diseases in which intestinal inflammation is difficult to distinguish from lymphoma, which is a neoplasm. In this study, the expression of the proteins S100A8/A9 and S100A12 (also called calgranulins) in the intestine is investigated in both diseases and for potential correlations with microscopically visible changes in the intestine or the clinical severity of the disease. Only small differences were seen between healthy and diseased animals, and there were no differences between cats with intestinal inflammation and lymphoma. However, several correlations of cells staining positive for calgranulins and inflammatory changes at the microscopic level and clinical disease severity were shown. This indicates that calgranulins play a role in both gastrointestinal lymphoma and inflammation and would support the recent theory that these two diseases might not be separate disease entities but instead are related. Further insights into the role of the calgranulins in these feline diseases will lead to a better understanding of the disease pathogenesis and, thus, potentially novel diagnostics and treatment avenues.

**Abstract:**

Diagnosing chronic inflammatory enteropathies (CIE) in cats and differentiation from intestinal lymphoma (IL) using currently available diagnostics is challenging. Intestinally expressed S100/calgranulins, measured in fecal samples, appear to be useful non-invasive biomarkers for canine CIE but have not been evaluated in cats. We hypothesized S100/calgranulins to play a role in the pathogenesis of feline chronic enteropathies (FCE) and to correlate with clinical and/or histologic disease severity. This retrospective case-control study included patient data and gastrointestinal (GI) tissues from 16 cats with CIE, 8 cats with IL, and 16 controls with no clinical signs of GI disease. GI tissue biopsies were immunohistochemically stained using polyclonal α-S100A8/A9 and α-S100A12 antibodies. S100A8/A9^+^ and S100A12^+^ cells were detected in all GI segments, with few significant differences between CIE, IL, and controls and no difference between diseased groups. Segmental inflammatory lesions were moderately to strongly correlated with increased S100/calgranulin-positive cell counts. Clinical disease severity correlated with S100A12^+^ cell counts in cats with IL (*ρ* = 0.69, *p* = 0.042) and more severe diarrhea with colonic lamina propria S100A12^+^ cells with CIE (*ρ* = 0.78, *p* = 0.021) and duodenal S100A8/A9^+^ cells with IL (*ρ* = 0.71, *p* = 0.032). These findings suggest a role of the S100/calgranulins in the pathogenesis of the spectrum of FCE, including CIE and IL.

## 1. Introduction

Feline chronic enteropathy (FCE) is one of the most common disorders in elderly cats, with an increasing prevalence over the last years [1,2]. While there are many studies on chronic enteropathies in dogs, much less is known about FCE [3,4,5]. Currently, FCE is defined as the presence of gastrointestinal (GI) clinical signs for ≥3 weeks and exclusion of other GI conditions (e.g., endoparasitic infections) and extra-GI diseases (e.g., hyperthyroidism) [3,6]. The most common types of FCE are chronic inflammatory enteropathy (CIE) and small-cell alimentary lymphoma (SCL) [6].

The etiopathogenesis of CIE has not been fully elucidated but appears to result from a complex interplay between environmental factors (e.g., food antigens, intestinal microbiome), a dysregulated immune response, and genetic predisposition [4,6]. The subclassification of CIE used in most investigations is retrospectively based on the clinical response to treatment into food-responsive enteropathy (FRE) and immunosuppressant-responsive enteropathy (IRE), which cannot necessarily be differentiated by histology [6,7]. Older literature also includes the CIE subclass antibiotic-responsive enteropathy (ARE), the existence of which in cats is debated and more likely presents an idiopathic or secondary small intestinal dysbiosis [8].

The three main types of feline GI lymphoma are low-grade SCL, intermediate-to-large-cell lymphoma (LCL), and large granular lymphoma (LGL). These subtypes vary in histologic appearance, treatment, and prognosis, whereby SCL accounts for approximately 75% of all GI lymphoma cases in cats. SCL is characterized by a well-differentiated population of small lymphocytes with low mitotic rates and usually slow clinical progression (median survival time [MST]: 19–29 months) [9]. In contrast, LGLs have an immature and less differentiated cell population, higher mitotic rates, and faster progression, leading to more rapid clinical courses (MST: 7–10 months) [10,11].

Currently, the subclassification of feline CIE as either FRE or IRE and the differentiation from diffuse infiltrative neoplasia (particularly SCL) is challenging [12]. SCL and CIE largely overlap concerning patient signalment (age, breed), clinical signs (weight loss, vomiting, anorexia, diarrhea), and the results of non-invasive diagnostics (routine blood work, abdominal ultrasonography) [5,6,13,14,15,16,17,18,19]. Histopathology is currently the most reliable diagnostic to document the presence of FCE [6]. The most common histologic type of feline CIE is lymphoplasmacytic enteritis (LPE), with the inflammatory infiltrate localized to the lamina propria (and in some cases, the intestinal epithelium) and often accompanied by architectural lesions of tissue. In SCL cases, epithelial, mucosal, or transmural infiltration with well-differentiated neoplastic lymphocytes is often accompanied by lymphoplasmacytic inflammation [20,21,22,23]. However, diagnosing LPE alone is not equivalent to confirming CIE and can also be associated with food hypersensitivity, intestinal parasites, and hyperthyroidism [1], which need to be excluded as differentials. Often, CIE and SCL cannot be distinguished based on histology alone, and advanced diagnostics, including immunohistochemistry (IHC) and polymerase chain reaction (PCR)-based methods, are needed [24,25]. A recent study also showed promise for the diagnostic utility of histology-guided mass spectrometry [26].

The mainstay of treatment for feline IRE and SCL cases involves immunosuppression or chemotherapy with corticosteroids (e.g., prednisolone), calcineurin inhibitors (cyclosporine), and/or alkylating agents (chlorambucil) [1,4,6], all of which can have significant adverse effects. More targeted pathway-specific treatment options would be desirable in the hands of the small animal practitioner. Thus, further evaluating the inflammatory pathways involved in FCE will improve our understanding of these conditions and potentially open new avenues to individualized therapeutic intervention.

Calprotectin, the S100A8/A9 protein complex, belongs to the damage-associated molecular pattern (DAMP) molecules of the innate immune response and is a fecal biomarker of intestinal inflammation in dogs [27,28,29,30,31,32]. It is expressed primarily by neutrophils and activated macrophages (MΦ) and also in epithelial cells [33,34,35] and presents a ligand for Toll-like receptor (TLR)-4, a key molecule in acute and chronic inflammatory processes [36]. The S100A12 protein, also referred to as calgranulin C, is also a DAMP molecule [33,34] and is primarily released from activated polymorphonuclear cells [33,34,35]. S100A12 can bind to several innate immune receptors, including the receptor for advanced glycation end products (RAGE) [37], with a central role in innate and acquired immune responses [27,38]. Concentrations of S100A8/A9 and S100A12 in fecal samples are increased in dogs with CIE and correlate with the severity of clinical signs, endoscopic lesions, histologic changes, and disease subclassification [28,29,30,31]. Similar to dogs, the intestinal mucosal cellular infiltrates in cats with CIE are predominated by lymphocytes, plasma cells, and eosinophils, whereas neutrophils seem to represent only a small proportion of cells [3,4,39,40]. The role of MΦ, particularly of the MΦ1/MΦ2 polarization, remains unclear in canine CIE [41,42] and has not been evaluated in feline CIE. Preliminary data in cats suggest that fecal calprotectin [43] and S100A12 [44] are also significantly increased with chronic GI inflammation, but the source of these S100/calgranulins in the GI tract in FCE remains unknown.

Our central hypothesis was that the S100/calgranulin proteins, S100A8/A9 and S100A12, play a role in the pathogenesis of FCE, particularly CIE, and that the expression of S100A8/A9 and S100A12 in GI tissue biopsy specimens is therefore increased (due to primary or tumor-associated inflammation) and reflects the number and/or activity of intestinal mucosal polymorphonuclear cells in cats with feline alimentary lymphoma and/or CIE compared to healthy controls. Further, we hypothesized that the expression of S100A8/A9 and S100A12 correlates with the severity of histologic lesions and clinical disease as assessed by the feline chronic enteropathy activity index (FCEAI [5]) score. Thus, our study aimed to evaluate S100A8/A9 and S100A12 expression in GI tissue biopsies from cats with FCE and healthy controls.

## 2. Materials and Methods

### 2.1. Ethics Approval

Ethics approval was not required for the cases enrolled in this study at the University of Leipzig College of Veterinary Medicine (UL-CVM), as patient data and archived sample materials were retrospectively included, and owners provided their written consent for the use of data and surplus materials from routine diagnostics on the standard patient admission form of the UL-CVM Department for Small Animals.

### 2.2. Study Population and Routine Diagnostics

**Figure 1:**
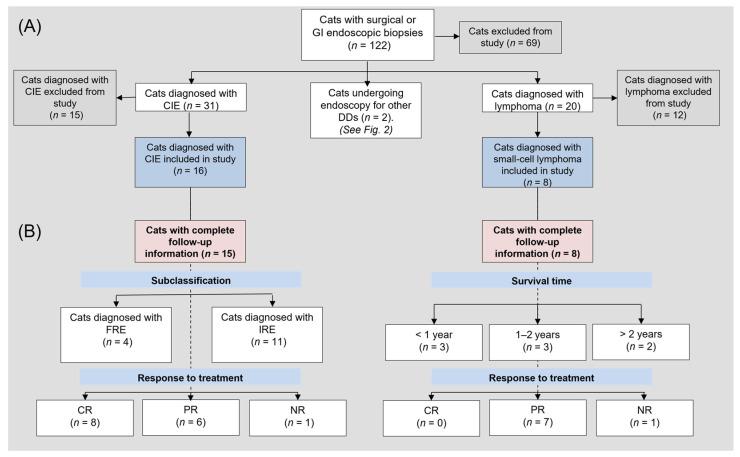
Flowchart of (**A**) all cats with chronic enteropathies included in the study and (**B**) those cats with FCE and complete follow-up information. Abbreviations: *n* = number; CIE = chronic inflammatory enteropathy; FRE = food-responsive enteropathy, IRE = immunosuppressant-responsive enteropathy; GI = gastrointestinal; DD = differential diagnosis; LCL = large-cell lymphoma; SCL = small-cell lymphoma; CR = complete remission; PR = partial remission; NR = no response. State of remission was based on clinical signs, partial remission in lymphoma cases was defined as “stable disease”.

Patient medical records of all cats that underwent GI endoscopy between 01/2011 and 01/2019 (*n* = 71) for various differentials and of all cats that had surgical or endoscopic GI biopsies for suspicion of CE (*n* = 51) at the UL-CVM Department for Small Animals were searched (*n* = 122) to identify all cats with a diagnosis of CIE or intestinal lymphoma, confirmed by histopathological examination, between January 2011 and July 2021. Complete medical records of cats with differentials CIE or IL (*n* = 53) were reviewed by one of the investigators (DR), and cats were selected for inclusion in the study based on (i) the presence of clinical signs for a minimum of 3 weeks prior to routine diagnostic evaluation and sample collection, (ii) exclusion of other underlying GI (e.g., endoparasites), extra-GI, or non-lymphoid neoplastic conditions, (iii) not receiving any medication that may interfere with calgranulin expression (e.g., corticosteroids [45] or non-steroidal anti-inflammatory drugs at the time of first biopsy), and (iv) a final diagnosis of either CIE or IL by a board-certified veterinary pathologist (Figure 1).

The possibility of other conditions was evaluated based on a minimum database, including a complete blood count, serum biochemistry profile, fecal examination by flotation (*n* = 23), and *Giardia* spp. antigen ELISA (*n* = 20), and abdominal ultrasonography. Sonographic findings were evaluated based on the presence of increased total GI wall thickness (>2.5 mm for the duodenum and jejunum and >3.2 mm for the ileum; level of increase graded as normal, mildly increased, or markedly increased [46]), loss of GI wall layering, increased thickness of the muscularis layer (>0.3 mm for the duodenum, >0.4 mm for the jejunum, and >0.9 mm for the ileum; graded as normal, mildly increased, or markedly increased [46]), enlarged mesenteric lymph nodes, and the presence of (scant) ascites [47].

If deemed to be indicated by the attending clinician, serum feline-specific pancreatic lipase (fPLI, measured as Spec fPL) (*n* = 20), cobalamin (*n* = 23), folate (*n* = 16), fructosamine (*n* = 18), total thyroxine (T4; *n* = 17), and feline trypsin-like immunoreactivity (fTLI) (*n* = 6) concentrations, urine analysis (*n* = 10), retrovirus (FeLV/FIV) status (*n* = 18), bile acid stimulation test (*n* = 3), and thoracic radiography or computed tomography scan (*n* = 22) were performed; 3 cats were tested for *Tritrichomonas foetus*. Further diagnostic evaluation of the cats included an upper (*n* = 6) or combined upper and lower (*n* = 13) GI endoscopy with mucosal biopsies or exploratory laparotomy with surgical (full-thickness) GI biopsies (*n* = 5). Endoscopy was repeated in 2 cats after 29 and 5 months, respectively.

The FCEAI score [5] was retrospectively calculated for all cats diagnosed with CIE or GI lymphoma to assess the clinical disease severity at the time of diagnosis. This 9-parameter scoring system considers the following parameters: GI endoscopic lesions, abnormal serum total protein (TP) concentration, ALT/ALP activity, phosphorous concentration, attitude/activity, and the presence of GI clinical signs, including vomiting, diarrhea, weight loss, and/or hyporexia. Clinical signs were graded from 0 to 3 depending on the severity (0 = normal, 1 = mild, 2 = moderate, 3 = severe), the remaining variables were dichotomously scored (0 = normal, 1 = increased (TP, ALT, ALP) or decreased (albumin, phosphorus)) [5]. Because no ranges have been established for interpreting the disease severity based on the cumulative FCEAI score, the following categories were considered: mild (FCEAI score of 0–5), moderate (FCEAI score of 6–12), and severe clinical disease (FCEAI score of 13–19).

A standardized questionnaire was used to obtain patient follow-up information from the owners and referring or attending veterinarians, asking for information regarding clinical signs, treatment, and individual outcomes. Based on this follow-up information, cats in the CIE group were subclassified as either FRE or IRE, and the survival times and response to treatment were analyzed.

**Figure 2:**
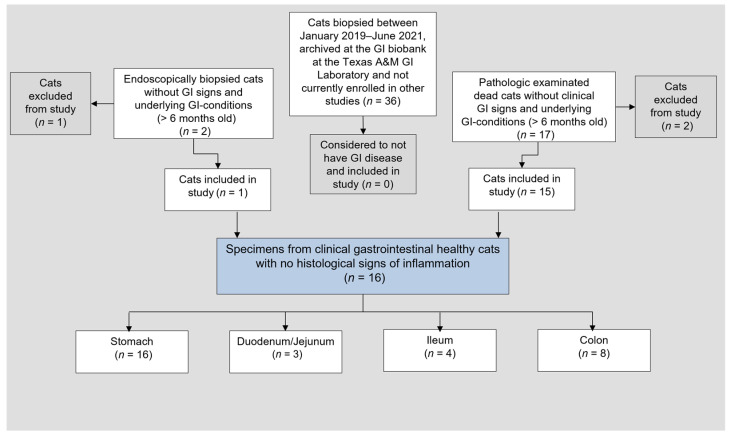
Flowchart of all cats included in the control group. Note that none of the cats was considered to be completely free of GI disease; thus, full-thickness tissue specimens of GI segments and areas with no histologic lesions were included as control tissues. GI = gastrointestinal.

For establishing a control group (Figure 2), medical records were also searched for cats without GI disease (*n* = 1) based on histopathology results as determined by a board-certified veterinary pathologist (WvB). Data and GI tissues of cats biopsied between January 2019–June 2021, archived at the Texas A&M University Gastrointestinal Laboratory and not currently enrolled in other studies, were also reviewed (*n* = 36), but none of these cats were considered as not having histologic evidence of GI disease. Tissue specimens from cats (*n* = 17) that were euthanized or died because of a non-GI-related cause at the UL-CVM Department for Small Animals or sent for autopsy to the UL-CVM Institute of Veterinary Pathology between April 2021–January 2022 with no prior history of GI signs or disease were obtained and examined by a nationally accredited veterinary pathologist (DB). Because none of the cats were considered free of any GI lesions in all segments of the GI tract, those full-thickness GI segments and areas with no histologic evidence of inflammation were included as control tissues.

### 2.3. Tissue Sample Collection and Routine Evaluation

A minimum of 5 endoscopic or between 1 and 3 full-thickness tissue biopsies per section and animal were obtained from the stomach (*n* = 35), duodenum/proximal jejunum (*n* = 27), ileum (*n* = 14), and/or colon (*n* = 18). These biopsies were used for routine histopathology and to evaluate the intestinal mucosal expression of S100A8/A9 and S100A12. Two cats diagnosed with GI lymphoma were re-biopsied when clinical signs recurred or worsened 29 and 5 months after the first diagnostic investigation of the cat; these biopsies were entered in the data analysis as individual tissue samples.

The tissues were submitted for routine histologic evaluation (WvB, DB) using the criteria of the WSAVA Gastrointestinal Standardization grading system [39] to assess morphologic lesions and inflammatory changes (including the severity of neutrophilic and macrophage infiltration) in the stomach, duodenum/proximal jejunum, ileum, and colon each on a 4-point scale (0 = normal, 1 = mild lesions, 2 = moderate lesions, and 3 = severe lesions). Cumulative lesion scores (i.e., the sum of individual lesion scores) were calculated. IHC analysis for CD3 (T cell) and CD20 positivity (B cell) was performed for all animals diagnosed with CIE or GI lymphoma (WvB, DB). For 18 of the cats, CD-staining was semi-quantitatively and spatially analyzed (DB) based on the criteria of Freiche et al. (2021) [48] (Supplementary Figure S1) with specific evaluation of villus atrophy, intraepithelial nests/plaques of lymphoid cells, lymphocytic cryptitis, cell counts and T-lymphocyte gradient in the lamina propria, infiltration of the intestinal epithelium, submucosa and subserosa/serosa with lymphoid cells, and percentage of CD3- and CD20-positive staining cells in the lamina propria and epithelium. Additionally, PCR for antigen receptor rearrangement (PARR) was performed on tissue biopsies from 10 cats at the Department of Pathobiology, University of Veterinary Medicine Vienna, Vienna, Austria.

### 2.4. IHC for Tissue S100A8/A9 and S100A12 Protein–Protocol Adjustment for Feline Tissues

**Figure 3:**
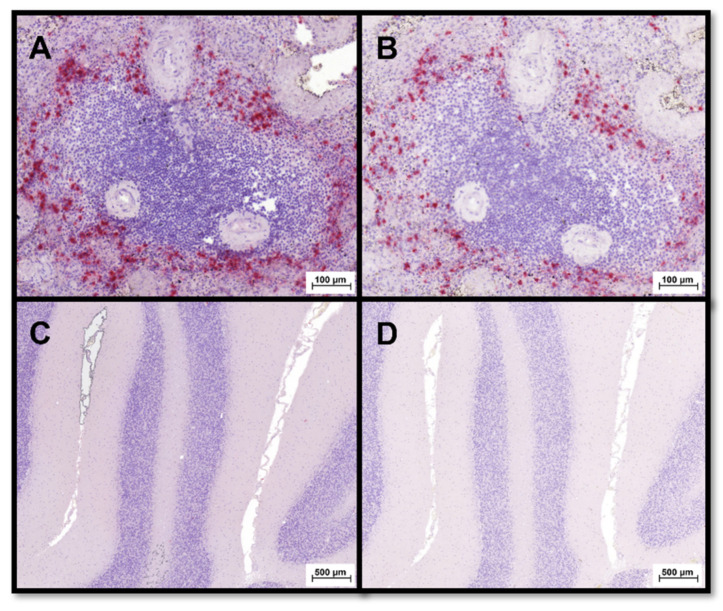
Evaluation of S100A8/A9- and S100A12-specific staining in feline tissues using polyclonal anti-canine S100A8/A9 and anti-S100A12 antibodies [35]. Large numbers of cells staining positive (red) for (**A**) S100A8/A9 and (**B**) S100A12 were detected in feline splenic tissue (used as positive control). In contrast, feline cerebral cortex (negative control) showed no cells staining positive for (**C**) S100A8/A9 nor (**D**) S100A12.

Polyclonal rabbit antibodies generated against canine S100A8/A9 [49,50] and canine S100A12 protein [51,52,53] were used for IHC analysis of S100A8/A9 and S100A12 expression. Cross-reactivity of these polyclonal antibodies with feline S100A8/A9 and feline S100A12 has been demonstrated in immunodiffusion experiments and xenospecies immunoassays employing these antibodies or antiserum [49,51,54,55]. Surplus materials provided by the autopsy service at the Institute of Veterinary Pathology at UL-CVM were used as IHC controls. Feline brain tissue served as a negative control, whereas feline spleen was used as a positive control (Figure 3).

All tissue specimens were fixed in neutral buffered formalin (10%) for a minimum of 24 h at room temperature (approximately 23 °C) and embedded in paraffin wax. The blocks containing the tissue biopsies were then cut into 2 μm or 3 μm thick sections for IHC and other analyses (e.g., CD3 or CD20 staining).

For S100A8/A9 and S100A12 staining, a modification of the IHC protocol originally established for tissues from dogs [35] was used. Briefly, following deparaffinization and rehydration with xylene and ethanol, each of the biopsy sections underwent antigen retrieval by heat treatment (95 °C) in 0.01 M citrate buffer (pH 6.0). Slides were then washed in phosphate-buffered saline (PBS), and nonspecific binding sites were blocked in PBS supplemented with 4% bovine serum albumin (BSA). After incubating the slides with the primary antibodies (purified polyclonal anti-canine S100A8/A9 [49] at 0.2 µg/mL and polyclonal anti-canine S100A12 [53] at 0.25 µg/mL in PBS with 4% BSA; normal rabbit serum at 0.2 µg/mL in PBS supplemented with 0.025% (*v*/*v*) Tween-20 (PBST) served as negative staining control), the secondary antibody (goat anti-rabbit alkaline phosphatase-labeled IgG, Dianova, Hamburg, Germany; diluted at 1 µg/mL in PBST) was added. Fast-red (Sigma-Aldrich, St. Louis, MO, USA) was used as a substrate, visualizing positive staining as a red product, and hematoxylin was added for counterstaining. After mounting the slides using Fluoromount™ aqueous mounting media (Sigma), standard light microscopy (AX70 microscope, Olympus, Centre Valley, PA, USA) was used to assess the quality of staining in the tissue biopsies.

### 2.5. S100A8/A9 and S100A12 IHC Analysis of Feline GI Tissue Biopsies

Tissue samples from 26 cats with FCE, of which 16 cats were diagnosed with CIE and 8 cats with alimentary lymphoma (2 cats biopsied twice), and 16 healthy controls were used for this study. All tissues were stained as described (Supplementary Figure S2).

All slides were converted to digital data using 3D Histech Pannoramic Scan II (3D Histech Ltd., Budapest, Hungary), and the images were analyzed using a free-ware histology slide viewing and evaluation software (SlideViewer v2.3, 3D Histech, Budapest, Hungary). A board-certified veterinary pathologist (CG) and trainee (DR) independently evaluated the number of positive-staining cells in all GI biopsies (blinded to the identity, clinical, clinicopathologic, and histopathologic results); these results were then combined, and a consensus was reached for any deviating cell counts that were obtained.

**Figure 4:**
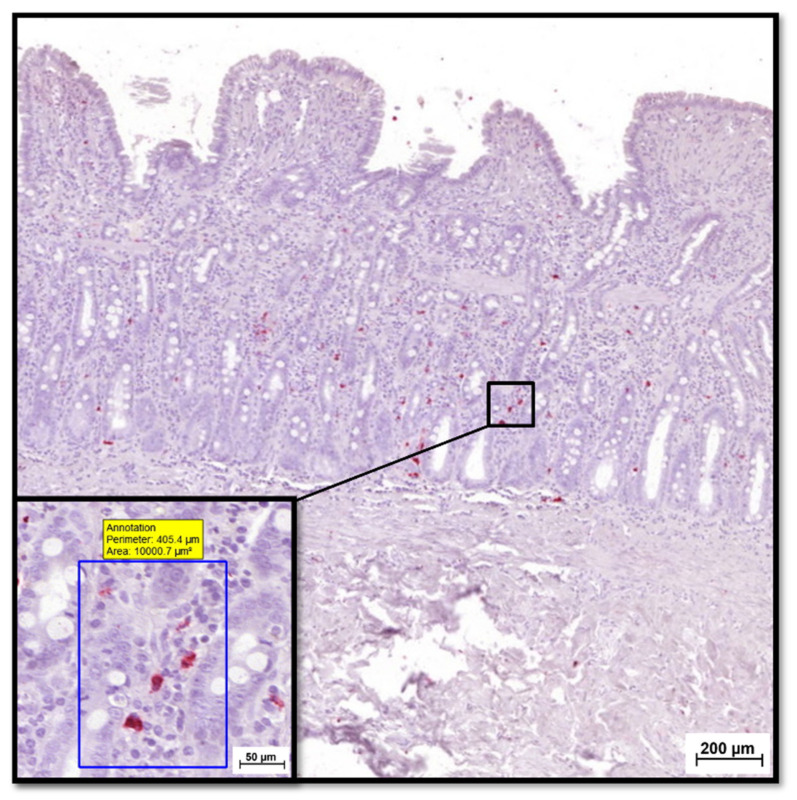
IHC for tissue S100/calgranulin protein expression. Shown is an example for selecting 1 of the 10 defined areas within the lamina propria (0.01 mm^2^ each, examined for each mucosal compartment and GI segment) of a duodenal endoscopic biopsy obtained from a cat with CIE.

**Figure 5:**
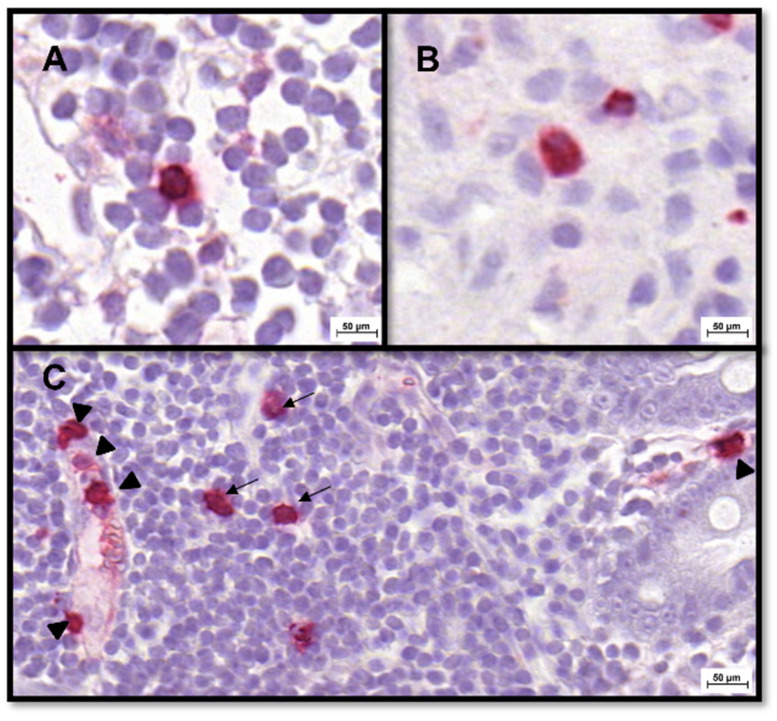
S100A8/A9- and S100A12-IHC of feline GI tissue biopsies. Numbers of cells staining positive for (**A**) S100A8/A9 and (**B**) S100A12 were evaluated. As demonstrated in (**C**), only infiltrating or resident cells staining positive for S100/calgranulins (arrows), but not intravascular cells (arrowheads), were counted and included in the analyses.

Mucosal S100A8/A9 and S100A12 positivity was assessed separately for the 2 different mucosal compartments: (i) the epithelial layer and (ii) the lamina propria. S100A8/A9^+^ and S100A12^+^ cell counts were determined for each segment (stomach, duodenum/proximal jejunum, ileum, and colon) and compartment (epithelium and lamina propria) (Figure 4) by counting all cells staining positive for S100A8/A9 or S100A12 in 10 defined areas (regions of interest that did not include intravascular cells, 0.01 mm^2^ each) under high-power fields (Figure 5). The mean number of positive cells identified in all fields was calculated and used for statistical analysis.

### 2.6. Statistical Analyses

Commercially available software packages (JMP^®^ v13.0, SAS Institute, Cary, NC, USA and GraphPad Prism^®^ v9.0, GraphPad Software, San Diego, CA, USA) were used for all statistical analyses. Based on the results of a similar study [35] in dogs, a sample size of 10 per group was expected to be sufficient. Normality of the data distribution was tested using a Shapiro–Wilk test, and the summary statistics were reported as medians and interquartile ranges (IQR) or ranges (for continuous data) or counts and percentages (for categorical data). Two-group or multiple-group comparisons were performed using a Wilcoxon rank-sum and Kruskal–Wallis test, respectively. A likelihood ratio test was performed to test for associations of categorical variables and correlation analyses by calculating a Spearman correlation coefficient (*ρ*). Statistical significance was set at *p* < 0.05, and a Holm-Bonferroni correction was applied for multiple correlations with the number of categories or subcategories considered.

## 3. Results

### 3.1. Patient Clinical Data

Cats with GI lymphoma were significantly older than those diagnosed with CIE (*p* = 0.039; Supplementary Table S1) or healthy controls (*p* = 0.016; age available for 14 healthy controls), with no difference between CIE and healthy control cats (*p* = 0.901). No differences were seen in the sex distribution (*p* = 0.340) or body weights (*p* = 0.368) between both disease groups of cats. Most cats in both groups were Domestic shorthair cats. Disease duration prior to presentation for diagnostic investigation and survival time after diagnosis did not differ between both groups (*p* = 0.254 and *p* = 0.364). Retrovirus status was negative in all cats that were tested. A total of 21 cats (13 cats with CIE, 7 IL cases, and 1 control cat) were tested for *Giardia* spp. and 3 cats (2 cats with CIE and 1 IL case) for *Tritrichomonas foetus*, with all being negative for either. Diagnostic imaging was pursued in all cats, with abdominal and thoracic radiographs performed in 15 cats with CIE and 7 cats with IL, and abdominal ultrasound with still images and video sequences available for re-review for all 24 cats. Sonographic findings did not differ significantly between cats with CIE and those with lymphoma, but compared to the latter, CIE cats tended to less frequently have sonographic evidence of ascites (25% vs. 60%; *p* = 0.075). Cats with CIE had significantly lower FCEAI scores than cats with lymphoma (*p* = 0.011), with less severe hyporexia (*p* = 0.039) and weight loss (*p* = 0.014; Supplementary Table S1).

### 3.2. Clinicopathologic Parameters

Hypocobalaminemia was significantly more frequently detected in cats with lymphoma compared to cats with CIE (88% vs. 44%, *p* = 0.045). No significant differences were seen in serum TP, albumin, globulin, total calcium, blood urea nitrogen (BUN), inorganic phosphorus, the prevalence of anemia (19% vs. 25%) and leukocytosis (38% vs. 50%), and serum fPL and fructosamine concentrations (all *p* > 0.05). Total T4 was within the reference interval in all cats, and serum fTLI concentrations (all > 16.4 μg/L) were determined in 2 cats with CIE and 4 IL cases. 

### 3.3. Endoscopy, Histopathology, and Clonality Testing

Endoscopy and sampling of biopsies were performed and documented (images and reports) in 19 cases (esophagogastroduodenoscopy combined with ileocolonoscopy in 13 cases and esophagogastroduodenoscopy alone in 6 cats; endoscopy was repeated after 29 and 5 months in 2 cats with IL) and surgical biopsies were obtained in 5 cats. For the control group, samples were obtained from 15 cats during autopsy, and 1 cat underwent esophagogastroduodenoscopy (Figure 2). Endoscopic lesions were detected in 11/13 cats (85%) with CIE and in 6/6 cats (100%) with IL (Supplementary Table S1). Linear hyperemic lesions were seen endoscopically in 1 cat, each with CIE and lymphoma.

Inflammatory lesion scores in the stomach, duodenum/proximal jejunum, and ileum were significantly lower in cats with CIE than in those with lymphoma (*p* = 0.026, *p* < 0.0001, and *p* = 0.019), which was not seen for the colon, and there were no differences in morphologic lesion scores between both patient groups. PARR showed polyclonality for TCRγ in 2 cats and bi- or oligoclonality (with a polyclonal background) in 3 cats with CIE, whereas mono-, bi-, or oligoclonality was detected in all 5 cats diagnosed with IL (and a polyclonal background in 2 of those cats). Helicobacter-like organisms (HLO) were present in gastric biopsies from 9 cats (47%), including 6 cats with gastric inflammation and 3 cats with normal gastric histology. The presence of HLO was not linked to gastric mucosal S100/calgranulin-positive cell counts or endoscopic lesions but was associated with less severe histologic lesions (*p* = 0.012) due to lower numbers of lymphocytes and plasma cells (*p* = 0.024).

### 3.4. Follow-Up and Response to Treatment

Long-term follow-up information was available from 15 cats (94%) with CIE and all 8 lymphoma cases (Figure 1). Response to treatment differed significantly between both disease groups (*p* = 0.011). In the CIE group, 8 cats (53%) achieved complete remission, 6 cats (40%) partially responded, and 1 cat (7%) had no clinical response. Four cats (27%) were diagnosed with FRE, of which 2 cats received a commercial novel protein (monoprotein) and 2 cats an easily digestible GI diet. The remaining 11 cats were classified as IRE based on the feeding of a GI or monoprotein diet (5 cats), hydrolyzed protein diet (1 cat), or both tried sequentially (4 cats), yielding only a partial or no clinical response but significant improvement or resolution of clinical signs on anti-inflammatory or immunosuppressive (prednisolone) treatment. Dietary intervention was not possible as part of the diagnostic evaluation in 1 cat classified as IRE. Additional treatments included an antiemetic (2 cats), prebiotic and/or probiotic (3 cats), supplementation of cobalamin (6 cats), pancreatic enzyme replacement therapy (1 cat), and antimicrobial therapy (1 cat).

**Figure 6:**
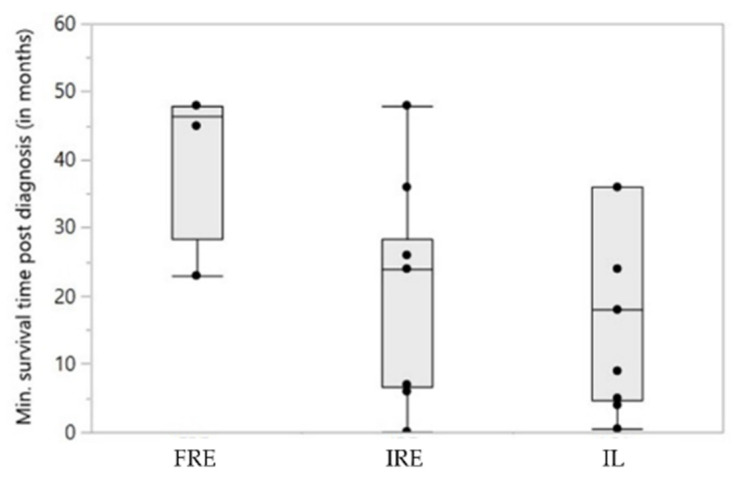
Survival times in cats with chronic enteropathy in this study. Cats diagnosed with chronic inflammatory enteropathies (CIE) survived longer (median: 24 months) after diagnosis than those with intestinal lymphoma (IL) (median: 18 months), but this difference was not significant. Within the CIE group, cats classified as food-responsive enteropathy (FRE) survived longer (median: 47 months) than those cats with immunosuppressant-responsive enteropathy (IRE) (median: 24 months), with no significant difference between both groups but significantly longer survival with FRE compared to IL (*p* = 0.027).

Most cats (88%) with lymphoma had improved clinical signs/stable disease on chemotherapy, with 1 cat (12%) experiencing progressive disease despite chemotherapy and 1 cat with an initial response relapsing after chemotherapy withdrawal. Chlorambucil was the most frequently administered chemotherapy drug, either as monotherapy (in 2 cats) or in combination with prednisolone (5 cats); 1 cat received a combination of prednisolone and cyclophosphamide. Progressive disease warranted including cyclophosphamide or doxorubicin (1 cat each) into the chemotherapy protocol. Additional treatments included a hydrolyzed protein diet (4 cats), an antiemetic (5 cats), supplementation of cobalamin (4 cats), pancreatic enzymes replacement therapy (2 cats), and antimicrobial therapy (1 cat). GI disease-associated death was less likely in cats with CIE (50%) than IL (100%; *p* = 0.012) with a relative risk (RR) of 0.5 (95% CI: 0.25–1.0). Survival times were longest in cats with FRE (median: 47 months) and differed significantly from cats with IL (median: 18 months, *p* = 0.027), but there was no significant difference compared to IRE (median: 24 months, *p* = 0.101; Figure 6). FCEAI scores at the time of diagnosis did not differ between FRE and IRE cats (*p* = 0.025).

### 3.5. Gastrointestinal S100/Calgranulin Expression

**Figure 7:**
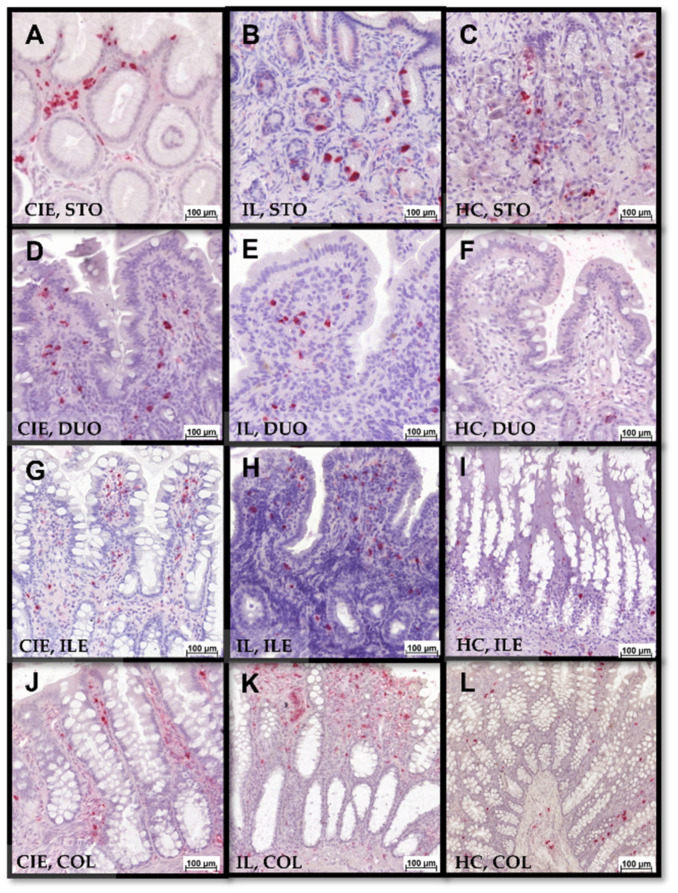
S100A8/A9-IHC of gastrointestinal tissue biopsies with varying numbers of intraepithelial and lamina propria S100A8/A9^+^ cells. Left to right: chronic inflammatory enteropathy (CIE), intestinal lymphoma (IL), GI healthy control group (HC); stomach (STO, **A**–**C**), duodenum (DUO, **D**–**F**), ileum (ILE, **G**–**I**), colon (COL, **J**–**L**). Note positively stained epithelial cells in (**B**).

**Figure 8:**
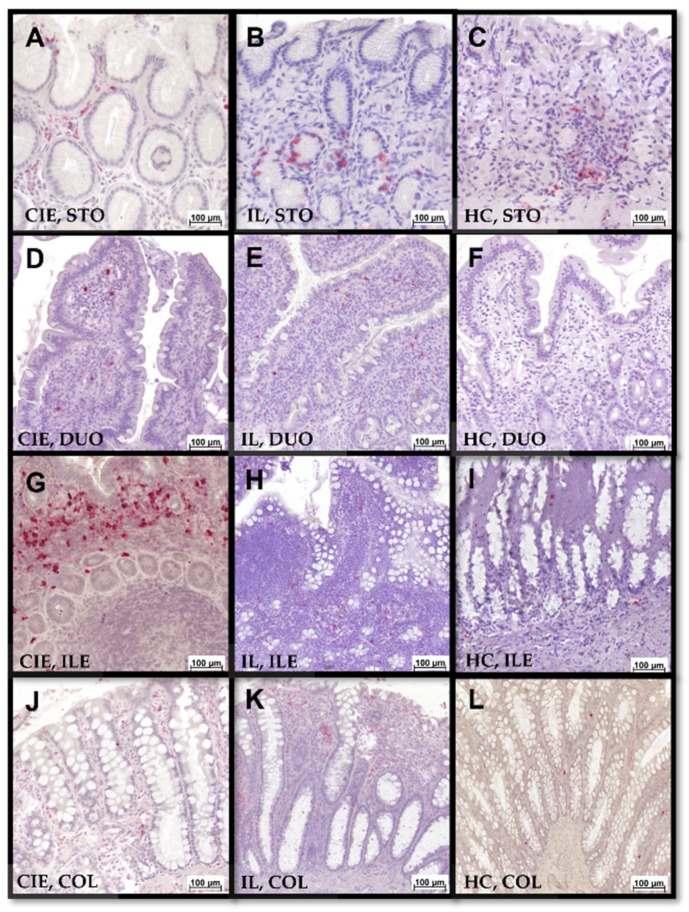
S100A12-IHC of gastrointestinal tissue biopsies with varying numbers of intraepithelial and lamina propria S100A12^+^ cells. Left to right: chronic inflammatory enteropathy (CIE), intestinal lymphoma (IL), GI healthy control group (HC); stomach (STO, **A**–**C**), duodenum (DUO, **D**–**F**), ileum (ILE, **G**–**I**), colon (COL, **J**–**L**). Note positively stained epithelial cells in (**B**).

Cats with FCE generally had slightly higher gastrointestinal mucosal S100A8/A9^+^ and S100A12^+^ cell counts than controls, albeit most comparisons were not statistically significant (Table 1 and Table 2). The numbers of S100A8/A9^+^ (Figure 7) and S100A12^+^ (Figure 8) cells in any of the GI segments evaluated were not significantly different between cats with CIE and cats with IL (all *p* > 0.05; Table 1 and Table 2) nor between cats with FRE or IRE (all *p* > 0.05), except for higher maximum numbers of S100 A8/A9^+^ cells in the gastric lamina propria in cats with FRE (median: 9, IQR: 3–13) than in IRE cats (median: 1, IQR: 0–7; *p* = 0.048). Strong correlations were detected between S100A12^+^ cell counts and the severity of lymphoplasmacytic infiltration, eosinophilic component, and the resulting inflammatory and cumulative histologic lesion scores in the colon (Table 3) of cats with CIE, whereas a similar pattern was detected as a trend for correlations with S100A8/A9^+^ cell counts. S100A8/A9^+^ cell counts were moderately positively correlated with the severity of follicular hyperplasia in the stomach (Table 3), whereas an inverse trend for a correlation with gastric S100A12^+^ cell counts was seen for intraepithelial lymphocytes. Finally, particularly S100A8/A9^+^ counts showed a trend for a positive correlation with ileal neutrophil and macrophage counts but an inverse relationship with the ileal cumulative histopathology score. No significant correlations or trends for associations with histologic lesions were seen for duodenal/proximal jejunal S100/calgranulin-positive cells.

Of note, a slightly positive (background) staining for S100A8/A9, but not S100A12, was invariably detected in some gastric cells (Supplementary Figure S3) and presumed to result from the reactions of parietal cells with peroxidase. Importantly, this cloudy background staining could be clearly differentiated from the positively stained cytoplasm of S100A8/A9^+^ and S100A12^+^ cells.

### 3.6. Association of Patient Characteristics with Gastrointestinal S100/Calgranulin Expression

S100/calgranulin-positive cell counts showed no association with the FCEAI scores in cats with CIE, but more severe diarrhea was associated with higher numbers of lamina propria S100A12^+^ cells in the colon (*ρ* = 0.78, *p* = 0.021). In cats with GI lymphoma, higher duodenal/proximal jejunal lamina propria S100A12^+^ cell counts correlated with higher FCEAI scores (*ρ* = 0.69, *p* = 0.042) and more severe diarrhea was linked to higher numbers of lamina propria S100A8/A9^+^ cells in the same GI segment (*ρ* = 0.71, *p* = 0.032). Given the small sample size, possible associations with colonic S100/calgranulin-positive cell counts could not be evaluated in cats with large intestinal lymphoma.

Serum cobalamin concentrations were positively correlated with the number of ileal epithelial S100A8/A9^+^ cells (*ρ* = 0.73, *p* = 0.008). This relationship and the same trend for lamina propria S100A8/A9^+^ and epithelial and lamina propria S100A12^+^ cell counts remained in the cats with CIE, whereas these trends were throughout inverse in cats with ileal lymphoma. Serum folate concentrations were not correlated with S100/calgranulin-positive cell counts in any of the GI segments evaluated (all *p* > 0.05). Cats with leukocytosis had higher duodenal/proximal jejunal numbers of S100A8/A9^+^ (median: 8.5 vs. 4; *p* = 0.012) and S100A12^+^ cells (median: 6.5 vs. 3; *p* = 0.014) than cats with normal peripheral leukocyte counts.

Lamina propria S100A12^+^ counts in the ileum were higher in hypoalbuminemic cats (median: 7.5) than in cats with normoalbuminemia (median: 1.5; *p* = 0.027). Compared to cats with normoglobulinemia, hyperglobulinemic cats had significantly higher duodenal/proximal jejunal S100A8/A9^+^ (median: 12 vs. 5; *p* = 0.006) and S100A12^+^ counts (median: 7 vs. 3; *p* = 0.016) and also higher ileal S100A8/A9^+^ (median: 11 vs. 3.5; *p* = 0.041) and S100A12^+^ counts (median: 8.5 vs. 1.5; *p* = 0.041). Endoscopic lesions in any GI segment were linked to higher duodenal/proximal jejunal lamina propria S100A8/A9^+^ counts (median: 8 vs. 1.5; *p* = 0.049), and some endoscopic lesions were linked to higher numbers of segmental S100/calgranulin-positive cells (Supplementary Table S2). Linear hyperemic lesions on endoscopy were rarely seen and could not be assessed for a relationship with S100/calgranulin-positive cell counts. There were also no differences in any segmental S100A8/A9^+^ or S100A12^+^ counts between cats with FRE and those classified as IRE.

## 4. Discussion

In this study, mucosal expression of S100A8/A9 and S100A12 in cats with CIE, GI lymphoma, and controls without GI lesions were evaluated and compared among groups as well as to the severity of clinical signs and histologic lesions. After careful scrutiny of the bibliographic databases, the authors believe that this is the first investigation of S100/calgranulin expression in cats with CIE and IL.

The results support our hypothesis that S100/calgranulins play a role in the pathogenesis of FCE, but their expression in GI tissue biopsies did not differentiate between CIE and IL. The lack of a correlation between numbers of mucosal S100/calgranulin-positive cells and polymorphonuclear cells on routine histology agrees with studies in dogs and could be explained by the plasticity of these cells. This plasticity of morphology can present a challenge for their routine detection and lead to misclassifications of the different types of polymorphonuclear cells. The presence of different grades of cellular infiltration, different stages of maturity, and inactive and activated cells during inflammation with spatiotemporal differences in their S100/calgranulin expression could be an alternative explanation [35,41]. S100/calgranulins are expressed in neutrophils and activated MΦ infiltrating the intestinal mucosa of patients with CIE [56]. More specifically, MΦ plays a central role in maintaining intestinal homeostasis and intestinal protective immunity. In the healthy intestines, MΦ predominantly serves an anti-inflammatory role, whereas increased recruitment of blood monocytes or activated resident cells differentiating into MΦ that display a proinflammatory phenotype are linked to inflammation [42,57,58]. Expression of the S100A8/A9 protein complex (L1 antigen) is restricted to immature proinflammatory MΦ in humans, and L1 expression is down-regulated during MΦ maturation [59]. Thus, increased L1^+^-MΦ counts reflect an influx of monocytes into the inflamed mucosa [35]. S100A8/A9 has been evaluated in dogs with CIE as a marker of early differentiated MΦ, whereby CD163 expression served as a marker for resident MΦ, showing decreased resident MΦ and increased early differentiated MΦ counts, and thus, more mucosal S100A8/A9^+^ cells in IRE and FRE. With S100A8/A9 expression in dogs presumed to decrease during the maturation of canine MΦ, the protein complex would be useful to detect migrating monocytes and early-stage MΦ rather than the resident MΦ population [60]. Based on these former results in dogs, it is likely that the same holds true for felines. The functional implications of increased intestinal S100A8/A9 expression in canine and feline CIE (e.g., link to TLR4 expression and/or responsiveness) require further research.

Few significant associations were seen for S100A12 expression in our study, with slightly more S100A12^+^ cells in the gastric lamina propria and colonic submucosa in the control group (Table 3). However, these results are based on overall low numbers of S100A12^+^ cells in addition to small group sizes. The results are consistent with previous findings in dogs, showing an inverse relationship between duodenal lamina propria MΦ and S100A12^+^ cell counts [35], with the lack of a correlation between fecal S100A12 abundance and the severity or presence of intestinal lamina propria phagocyte infiltration [29,30]. The hypothesis that the intestinal mucosa of dogs with CIE contains higher numbers of newly recruited (activated, proinflammatory) MΦ contributing to chronic inflammation [35] and expressing the S100A12 protein [58,61,62], whereas the MΦ population detected on routine histopathology is predominantly mature (anti-inflammatory, tissue-resident anergic) with reduced or absent S100A12 expression [62], is also reasonable in cats. Another theory is based on detecting higher Casp3 levels reflecting apoptotic activity in dogs with CIE compared to healthy controls [63], which may account for a shorter lifespan and lower S100A12 expression levels in infiltrating cells in CIE. Our findings are also in line with a study in human medicine, demonstrating higher S100-positive MΦ and neutrophil counts in non-inflamed than inflamed intestinal mucosa in patients with inflammatory bowel disease [64]. The association with inflammation is also supported by the higher numbers of S100/calgranulin-positive cells in cats with leukocytosis. Proinflammatory MΦ playing a role in CE is further supported by a study in dogs where a decrease in MHC II^+^, Iba-1^+^, CD204^+^, and CD162^+^ cells was seen in the duodenum and overall less MΦ in all GI segments [42].

However, this hypothesis requires further research in cats and dogs, and the exact population and activation status of S100A8/A9- and S100A12-expressing cells in feline CIE and IL remain to be studied. In addition, numbers of S100/calgranulin-positive cells were analyzed in this study but not the intensity of staining. A standardized, automated method determining the intensity of IHC staining could have quantified the mucosal S100/calgranulin expression but not distinguished intravascular cells from infiltrating or resident cells of the GI mucosa. Using a manual approach, only cells outside the intestinal blood vessels staining clearly positive for S100A12 or S100A8/A9 were considered and counted, but there was also some staining for both in the surrounding tissue outside defined cellular margins, which agrees with the findings of a previous study in dogs [35] and suggests secretion and localization of S100/calgranulins to the extracellular matrix [64]. Subjectively, different signal intensities for S100A8/A9 and S100A12 were noted in individual cats, which might result from higher expression levels in activated compared to non-activated cells.

The lack of correlation of the S100/calgranulin expression between cats with CIE and IL supports the recent proposal that CIE and alimentary lymphoma in cats might present different stages along the continuum or spectrum of the same disease process, in which CIE cases progress to SCL over time initiated by a chronic stimulation by a food or bacterial antigen or (unknown) viral pathogen [6,13,18,48,65]. This hypothesis of a continuum also exists in human celiac diseases that are suggested to progress from low-grade epitheliotropic lymphoma instead of inflammatory processes [66,67]. Alternatively, CIE and SCL might also be two distinct processes, and the inflammatory component in SCL could present a secondary process or an anti-tumor reaction.

It proved very difficult to identify cats that did not have any histologic lesions in any of the GI segments, especially in the duodenum and ileum. This agrees with a recent investigation of gastric and duodenal endoscopic biopsies from 20 clinically healthy cats [68]. With a median age of 9.5 years (range: 3–18 years), the demographic characteristics of that feline population were similar to the cats presenting with FCE in our study. Every cat in this previous study had abnormal histologic findings based on the WSAVA criteria. After a median follow-up time of 709 days, only 3 of the 20 cats (15%) developed GI signs, whereas the remaining 17 cats (85%) remained free of any clinical signs of GI disease during the follow-up period of the study (median: 709 days) [68].

The definition of a “normal intestinal microarchitecture” remains challenging in cats despite the WSAVA GI Standardization Group’s attempt to standardize the histologic assessment of GI biopsies with a grading scheme, evaluating cellular infiltration (e.g., lymphocytes/plasma cells, eosinophils, neutrophils, MΦ) and morphologic lesions (e.g., epithelial injury, crypt distention, lacteal dilation, villus blunting, fibrosis) on a scale from 0 (normal) to 3 (severely abnormal) [3,39]. The results of different observers still vary, and some investigators suggest simplifying the WSAVA grading scheme [47]. Cats defined as “normal” and used to establish the WSAVA guidelines were approximately 1.5 years old, specific pathogen-free (SPF) colony cats, which are hardly representative of the typical cat presented with GI signs in clinical practice. Thus, the definition of “GI healthy” may need to be revised, and more studies, including clinically healthy cats with no signs of GI disease and long-term outcomes (ideally followed until the time of death or euthanasia), would be very useful to establish a more realistic “healthy control” reference for future comparison.

We could not include healthy cats biopsied without a diagnostic and/or therapeutic indication for ethical reasons. Thus, the tissue samples from all cats in the control group (except for one cat) were obtained post-mortem, making autolysis and other post-mortem artifacts more likely than in the disease groups of cats. All control cats died or were euthanized for reasons other than GI diseases. However, none of the cats were completely healthy, and the underlying (non-GI) condition (e.g., cardiac failure, resulting in central venous congestion and intestinal dysfunction [69], spinal trauma potentially causing intestinal injury) could have contributed to higher intestinal S100/calgranulin-positive cell counts. Furthermore, during the process of dying, an intestinal stress response due to local ischemia accompanied by an acute inflammatory response with the recruitment of neutrophils, macrophages, and other immune cells may occur [70].

In addition, post-mortem examination in the control cats offered the entire GI tract to be examined and generally larger (full-thickness) specimens than those in the FCE groups, where sampling was restricted to certain areas of the GI segments.

Cats with FRE generally had better outcomes than those with IRE and significantly longer survival times than cats with lymphoma. Despite the small number of animals with FRE in this study (cats that respond to diligent dietary trials are rarely biopsied), this result should alert clinicians to always perform an elimination diet trial in cats with CIE before initiating immunosuppressive treatment. No difference in S100A8/A9^+^ and S100A12^+^ cells was seen between both CIE groups, except for a significantly higher maximum S100A8/A9^+^ cell count in the gastric lamina propria in cats with IRE.

Cats with CIE were younger than cats with GI lymphoma, although an overlap in the age ranges was noted, and these ranges agree with previous reports [5,13,14,15]. Similar to other studies, Domestic shorthair cats were overrepresented in both groups, likely due to being the most common feline breed seen at UL-CVM. Clinical signs have been reported to be indistinguishable between cats with GI lymphoma and those with CIE, with the most common clinical signs in both groups being weight loss (prevalence: 80–90%), followed by vomiting (70–80%), anorexia (60–70%), and diarrhea (50–65%) [16,17,18].

We hypothesized intestinal S100/calgranulin expression to correlate with the severity of histologic lesions and clinical disease. This was partially confirmed as S100A12^+^ cell counts correlated positively with the severity of lymphoplasmacytic and eosinophilic infiltration and the resulting inflammatory and cumulative histologic lesion scores in the colon, with similar trends seen for S100A8/A9^+^ cells. This finding agrees with the previously reported correlation of S100A12 levels in colonic mucosal tissue with the severity of overall histologic lesions and epithelial injury in the canine colon [71]. FCEAI scores and the severity of weight loss were significantly higher in cats with lymphoma than in those with CIE. Together with more severe inflammatory lesions in the duodenum/proximal jejunum in cats with lymphoma, this may also support the theory of a spectrum of diseases in which clinical signs progressively worsen over time, and the transition from inflammation to lymphoma leads to emaciation of the affected cat.

FCEAI scores did not correlate with S100/calgranulin expression in the CIE group, but cats with marked diarrhea had more colonic S100A12^+^ cells, similar to S100A8/A9^+^ cell counts in cats with IL. FCEAI scores in cats with IL also correlated with S100A12^+^ cell counts in the duodenum, suggesting a link between clinical disease severity and S100/calgranulin expression. The lack of an association between the FCEAI score and intestinal mucosal S100A12 positivity in cats with CIE is consistent with findings in dogs [35,71] and, again, the presence of select MΦ populations [42]. Correlation of segmental S100/calgranulin expression with clinical data (e.g., leukocytosis, endoscopic lesions) is also reported in human patients with IBD [72,73,74]. From a clinical perspective, it remains to be determined if intestinal S100/calgranulin expression reflects the fecal concentrations of these molecules. The presence of some staining for S100A8/A9 and/or S100A12 in the surrounding tissue outside defined cellular margins could indicate a release of inflammatory cells secreting S100/calgranulins to the extracellular matrix [64] and therefore leading to potentially higher S100/calgranulin levels in the feces [44] compared to intestinal tissue.

Histologic differentiation of severe LPE (CIE) from SCL can be challenging and often requires IHC and, in some cases, PCR-based methods (PARR) to confirm the diagnosis [6,75]. IHC employs specific stains (e.g., against CD3 for T-cells and CD20 for B-cells) to determine whether the infiltrating lymphocytes are of a single lineage, supporting a diagnosis of SCL. Infiltrates equally consisting of T and B cells support a diagnosis of CIE. However, SCL is often accompanied by inflammation, and even with IHC, establishing a diagnosis can be difficult [6]. Currently, PARR performed on formalin-fixed paraffin-embedded tissue samples is applied to determine the clonality of lymphocyte receptors [23]. Polyclonality (diverse receptors) on PARR suggests inflammatory lesions, whereas clonality (uniform receptors) indicates neoplasia. Because cell lineages cannot be determined by PARR, IHC and PARR analysis must be interpreted together and in the context of all diagnostics performed [6]. Histology and, in some cases, IHC for CD3/CD20 and PARR were performed and interpreted by several pathologists (CG, WvB, DB) blinded to each other and for CD-IHC and PARR also to the identity, clinical, clinicopathologic, and histologic results. Moderate to high inter-observer variance between different pathologists has been described [76,77], but very small discrepancies occurred in our study. Thus, all pathologic diagnoses were confirmed, except for two cases diagnosed by the pathologists’ consensus. This included a cat in the CIE group and a healthy control cat. In addition, the results of the PARR analysis in our study supported previous investigations questioning the reclassification of CE cases in cats based on clonality testing alone [5,11,48,78].

Due to the retrospective nature of this study and to evaluate a realistic study population similar to that expected in clinical practice, we did not limit IL cases to include SCL but also two cases of LCL. No differences were seen between SCL and LCL, but the statistical power to detect any differences between both was low. One of the cats with IL was re-biopsied 5 months after the first endoscopy and was diagnosed with LCL after an initial diagnosis of SCL. The first endoscopy diagnosed SCL in the duodenum, whereas the gastric mucosa showed only inflammation. This had progressed to lymphoma in all examined segments at the time of reevaluation, suggesting the potential for (possibly rapid) worsening of this condition and potentially supporting the theory of a disease spectrum of FCE characterized by the transition from LPE (CIE) to GI lymphoma. However, this warrants further studies. In addition, one cat of the lymphoma group diagnosed with duodenal LPE showed lymphoma in the ileum, which is the most common location for SCL [9] and should always be examined if lymphoma is suspected. For various reasons, we did not have tissue samples from all segments of the GI tract in all cats. Thus, we cannot exclude the possibility of misclassification of cats as CIE with “hidden” lymphoma in the ileum (if ileal biopsies were unavailable) or segments of the GI tract outside the reach of the endoscope. In addition, missing lymphoma in any GI segment during sampling cannot be completely ruled out. However, the study focused on the S100/calgranulin expression in the GI tract, where each GI segment was considered separately for analysis, leveraging this limitation. Still, it warrants reminding clinicians of the importance of obtaining tissue biopsies from all GI segments within reach of the endoscope (i.e., stomach, duodenum/proximal jejunum, ileum, and colon) for a reliable diagnosis. In addition, multiple tissue biopsy samples should be obtained from each GI segment, especially the ileum, to optimize the histologic assessment and reduce the risk of false results due to variation (e.g., patchy distribution of the mucosal disease process). It is generally recommended to obtain at least 6–8 adequate endoscopic biopsy samples per GI segment [79,80]. This number was not reached in all endoscopically obtained tissue samples, particularly due to using surplus material. Even fewer biopsies (max. 1–2 per segment, except for the colon) are obtained with a surgical approach, as was the case for 6 cats in this study. The lack of surgical colonic biopsies (usually obtained via colonoscopy) reflects the very high risk of suture dehiscence and colonic wall perforation after full-thickness incision of the large intestine in cats [81], which should always be weighed against the expected benefit for the diagnostic evaluation.

Due to the retrospective nature of this study, the patient medical data were incomplete in some cases, and the diagnostic evaluation, individual case management, and times of follow-up evaluations varied. In four cats, one FCEAI parameter (serum phosphorus concentration and serum ALP activity in 2 cats each) was not available and thus entered as “0” for FCEAI calculation. This was considered reasonable, given that, if at all, it could only have changed the cumulative FCEAI score by one point and would not have misrepresented the score for any of those four cats. As an advantage of the retrospective study design, a more reliable classification into the subgroups IRE, FRE, and lymphoma, which is usually challenging, was possible with the resulting long follow-up times and detailed information about the clinical course of the disease, response to treatment, and monitoring for any long-term consequences and/or other conditions. Still, some potential confounding factors cannot be excluded for all cats. In one cat, an intoxication (unknown toxic agent) could not be ruled out as a cause of worsening clinical signs. However, this cat had a chronic history of GI signs and laboratory, sonographic, endoscopic, and histopathologic findings, and the response to treatment was consistent with a diagnosis of IRE. Another cat (long-haired breed) was presented with acute ileus and a history of recurrent constipation and vomiting. After the endoscopic removal of a hairball, a dietary change to a commercial “anti-hairball” elimination diet, lactulose, and daily grooming led to clinical remission. Together with histopathology and CD3/CD20-IHC, this cat was classified as FRE. In this case and another cat with ileus as a primary cause for immediate endoscopy, the clinical signs just prior to the acute incidence were considered for calculating the FCEAI score. Interestingly, one cat in the CIE group responded well long-term to the combination of an antibiotic (metronidazole) and a probiotic for 4 weeks. While this might implicate a diagnosis of ARE, the cat also received an elimination diet, and the owners reduced several stress factors, resulting in the definitive classification as FRE because the dietary intervention was considered the main part of therapy in this cat.

Achieving remission in this study was based on clinical signs and non-invasive diagnostics alone because reinvestigation with endoscopy is often not performed even after unsuccessful treatment. This is a disadvantage of using the FCEAI score, which incorporates results of the cat’s endoscopic evaluation, and calls for a new (i.e., simplified) scoring system for FCE that allows for reevaluating cats without needing repeated endoscopy (similar to the currently used canine inflammatory bowel disease activity index [82] or canine chronic enteropathy clinical activity index [83]). Finding higher S100/calgranulin-positive cell counts linked to higher serum cobalamin concentrations was an unexpected finding of this study. We speculate this to reflect dysregulations in cobalamin metabolism in FCE, which is currently subject to further investigation by our group.

We acknowledge that this study has some further limitations. Due to the clinical character of this study, randomized sampling and stereologic analysis could not be applied. Additionally, specific staining was not performed to further characterize the cells expressing S100A12 and/or S100A8/A9. Thus, it cannot be determined if the expression of these proteins is localized to neutrophils, dendritic cells, and/or different types of MΦ in feline CE. Furthermore, the control group consisting of mostly autopsied cats was not ideal, and further evaluation of healthy cats would have been preferable but was not feasible for ethical reasons. Lastly, the sample size was small, and a type II error for finding no difference or association cannot be excluded.

Further studies are warranted to further characterize the population of cells expressing S100/calgranulins, for example, via double-staining for S100/calgranulins and polymorphonuclear cells and macrophages (particularly the proinflammatory phenotype), evaluate serial biopsies, and phenotypically characterize S100/calgranulin-positive stained cells. Stratification of the patient groups based on the level of inflammation and examination of fecal S100/calgranulin levels in cats are also needed.

## 5. Conclusions

In summary, the findings of this study support that S100A8/A9 and S100A12 do play a role in the pathogenesis of FCE and do not differ between the two main groups of FCE, which supports the theory of CIE and SCL presenting different stages along a spectrum of the same disease process. Additional studies are warranted to characterize further the population of cells expressing S100A8/A9 and/or S100A12 and to determine the functional implications of the differential expression of these proteins and their possible potential for diagnostic or monitoring purposes in FCE.

## Figures and Tables

**Table 1:** Gastrointestinal tissue S100A8/A9 positivity in the cats included in the study (*n* = 40).

		Epithelial S100A8/A9 Positivity (Cell Counts)			Lamina Propria S100A8/A9 Positivity (Cell Counts)			Submucosal S100A8/A9 Positivity (Cell Counts)		
Histologic Lesion	*n*	Range in All Tissue Biopsies	Median (Range)	*p*	Range in All Tissue Biopsies	Median (Range)	*p*	Range in All Tissue Biopsies	Median (Range)	*p*
Stomach	34									
normal	19	0–1	**0 (0) ^A^**	**0.011**	0–10	1.5 (0–5)	0.326	−	−	–
inflammation	13	0–10	**0 (0–4) ^B^**		0–13	1.5 (0.5–3)		−	−	
lymphoma	2	0–3	**1 (0–1) ^B^**		0–3	0.5 (0.5–1)		–	–	
Duodenum/Jejunum	27									
normal	2	0–2	0 (0–0.5)	0.837	0–5	**1.5 (0.5–2.5) ^A^**	**0.045**	0 *	0 (0) *	0.749
inflammation	16	0–12	0 (0–2)		0–38	**6 (0–21.5) ^A,B^**		0–6 ^$^	0 (0–1) ^$^	
lymphoma	9	0–6	0.5 (0–0.5)		0–27	**6.5 (3–12) ^B^**		0–8 ^†^	0 (0–2.5) ^†^	
Ileum	14									
normal	2	0	0 (0)	0.271	2–13	6 (3–9)	0.901	0–1	0.5 (0–0.5)	0.719
inflammation	8	0–1	0 (0–0.5)		0–24	3.5 (0.5–12)		0–4	0.5 (0–1)	
lymphoma	4	0	0 (0)		0–16	5 (3.5–7.5)		0–1 ^‡^	0.5 (0–0.5) ^‡^	
Colon	17									
normal	7	0	0 (0)	0.222	0–19	6.5 (2.5–8.5)	0.239	0–11	**1.5 (0.5–2.5) ^A^**	**0.004**
inflammation	9	0–2	0 (0–0.5)		0–23	4 (1–10.5)		0–1	**0 (0–0.5) ^B,§^**	
lymphoma	1	0	0 (0)		0–21	10 (10)		0–2	**0.5 (0.5) ^A,B^**	

Note: number of cats. * Available from *n* = 1 cat; ^$^ available from *n* = 10 cats; ^†^ available from *n* = 5 cats; ^‡^ available from *n* = 3 cats; ^§^ available from *n* = 7 cats. Parameters in bold font and green indicate significant differences at *p* < 0.05, where values stacked within a column that are not sharing a common superscript letter (A, B) are significantly different at *p* < 0.05.

**Table 2:** Gastrointestinal tissue S100A12 positivity in the cats included in the study (*n* = 40).

		Epithelial S100A12 Positivity (Cell Counts)			Lamina Propria S100A12 Positivity (Cell Counts)			Submucosal S100A12 Positivity (Cell Counts)		
Histologic Lesion	*n*	Range in All Tissue Biopsies	Median (Range)	*p*	Range in All Tissue Biopsies	Median (Range)	*p*	Range in All Tissue Biopsies	Median (Range)	*p*
Stomach	34									
normal	19	0–1	0 (0)	0.102	0–17	**1 (0–6) ^A^**	**0.032**	−	−	–
inflammation	13	0–10	0 (0–1)		0–6	**0.5 (0–2) ^B^**		−	−	
lymphoma	2	0	0 (0)		0–1	**0.5 (0–1) ^A,B^**		–	–	
Duodenum/Jejunum	27									
normal	2	0	0 (0)	0.153	0–7	1.5 (0.5–3)	0.342	0	0 (0) *	0.574
inflammation	16	0–13	0.5 (0–3.5)		0–23	4 (0.5–11)		0–2	0 (0–0.5) ^$^	
lymphoma	9	0–1	0 (0–0.5)		0–23	3.5 (1.5–8)		0–2	0 (0–0.5) ^†^	
Ileum	14									
normal	2	0	0 (0)	0.544	0–10	4 (2–6)	0.852	0–1	0.5 (0–0.5)	0.635
inflammation	8	0–1	0 (0–0.5)		0–17	2.5 (0.5–10)		0–1	0.5 (0–0.5)	
lymphoma	4	0–1	0 (0–0.5)		0–30	3 (0.5–7)		0–1 ^‡^	0.5 (0–0.5) ^‡^	
Colon	17									
normal	7	0	0 (0)	0.641	0–30	6 (0.5–11)	0.599	0–22	**0.5 (0–4) ^A^**	**0.041**
inflammation	9	0–3	0 (0–1)		0–24	3 (0.5–9)		0–1 ^§^	**0 (0–0.5) ^B,§^**	
lymphoma	1	0	0 (0)		0–12	5.5 (5.5)		0–2	**0.5 (0.5) ^A,B^**	

Note: number of cats. * Available from *n* = 2 cats; ^$^ available from *n* = 10 cats; ^†^ available from *n* = 5 cats; ^‡^ available from *n* = 3 cats; ^§^ available from *n* = 7 cats. Parameters in bold font and green indicate significant differences at *p* < 0.05, where values stacked within a column that are not sharing a common superscript letter (A, B) are significantly different at *p* < 0.05.

**Table 3:** Correlation of S100A12 and S100A8/A9 positivity with histologic findings in feline CIE. Shown are the correlations among epithelial and the lamina propria S100A8/A9^+^ [and S100A12^+^] cell counts and the severity of morphologic and inflammatory histologic lesions in the stomach, duodenum, ileum, and colon in cats with inflammatory lesions in these segments (*n* = 20).

Parameter	Correlated with	Spearman *ρ* Correlation Coefficient (*p*-Value, *P_corr_*)			
		Epithelial S100A8/A9^+^ (Cell Counts)	Lamina Propria S100A8/A9^+^ (Cell Counts)	Epithelial S100A12^+^ (Cell Counts)	Lamina Propria S100A12^+^ (Cell Counts)
		Stomach (*n* = 13)		Stomach (*n* = 13)	
Stomach (composite score) ^#^		0.14 (0.655)	0.08 (0.801)	0.06 (0.844)	0.33 (0.279)
Morphologic criteria (sum)		0.49 (0.092)	−0.16 (0.609)	0.38 (0.199)	0.18 (0.559)
- Surface epithelial injury		N/A	N/A	N/A	N/A
- Gastric pit epithelial injury		N/A	N/A	N/A	N/A
- Fibrosis/glandular nesting/MA		0.33 (0.916)	−0.26 (0.394)	0.21 (0.494)	−0.09 (0.780)
Inflammatory criteria (sum)		−0.02 (0.948)	0.25 (0.403)	−0.08 (0.791)	0.38 (0.206)
- Intraepithelial lymphocytes		−0.36 (0.221)	0.12 (0.689)	**–0.54** (0.059)	−0.25 (0.420)
- Lamina propria LPC		−0.01 (0.965)	0.19 (0.528)	−0.18 (0.555)	0.42 (0.155)
- Lamina propria eosinophils		N/A	N/A	N/A	N/A
- Lamina propria neutrophils		0.27 (0.380)	0.12 (0.705)	0.47 (0.104)	0.31 (0.303)
- Lamina propria ΜΦ		0.27 (0.380)	0.12 (0.705)	0.47 (0.104)	0.31 (0.303)
- Lymphofollicular hyperplasia		**0.59 (0.034; 0.204)**	0.06 (0.852)	0.28 (0.357)	0.32 (0.295)
		Duodenum/proximal jejunum (*n* = 16)		Duodenum/proximal jejunum (*n* = 16)	
Duodenum/proximal jejunum (composite score)		0.09 (0.747)	0.18 (0.515)	−0.08 (0.766)	0.13 (0.643)
Morphologic criteria (sum)		−0.02 (0.942)	0.11 (0.679)	−0.14 (0.614)	0.12 (0.671)
- Villus stunting		−0.03 (0.927)	0.16 (0.546)	−0.06 (0.837)	0.14 (0.594)
- Epithelial injury		−0.04 (0.880)	0.13 (0.632)	0.12 (0.667)	0.27 (0.320)
- Crypt distension		0.07 (0.795)	−0.23 (0.401)	−0.06 (0.816)	−0.22 (0.423)
- Lacteal dilation		−0.29 (0.289)	0.41 (0.115)	−0.42 (0.103)	0.21 (0.446)
- Mucosal fibrosis		0.02 (0.931)	0.08 (0.763)	−0.19 (0.481)	0.25 (0.359)
Inflammatory criteria (sum)		0.34 (0.196)	0.44 (0.087)	0.19 (0.474)	0.29 (0.282)
- Intraepithelial lymphocytes		0.12 (0.672)	0.06 (0.822)	−0.13 (0.620)	−0.13 (0.623)
- Lamina propria LPC		0.14 (0.619)	0.23 (0.395)	0.01 (0.991)	0.14 (0.619)
- Lamina propria eosinophils		0.10 (0.719)	0.26 (0.333)	0.22 (0.418)	0.17 (0.529)
- Lamina propria neutrophils		0.42 (0.102)	0.41 (0.115)	0.25 (0.344)	0.45 (0.080)
- Lamina propria ΜΦ		0.23 (0.402)	0.14 (0.605)	0.03 (0.916)	0.20 (0.467)
		Ileum (*n* = 8)		Ileum (*n* = 8)	
Ileum (composite score)		**–0.70** (0.053)	−0.34 (0.399)	**−0.54** (0.168)	−0.35 (0.399)
Morphologic criteria (sum)		**−0.58** (0.132)	−0.39 (0.346)	**−0.61** (0.112)	−0.28 (0.506)
- Villus stunting		−0.43 (0.284)	0.06 (0.882)	−0.44 (0.280)	−0.06 (0.882)
- Epithelial injury		−0.43 (0.289)	−0.02 (0.971)	0.05 (0.899)	0.09 (0.826)
- Crypt distension		−0.23 (0.585)	**−0.55** (0.157)	**−0.61** (0.111)	−0.35 (0.395)
- Lacteal dilation		−0.28 (0.496)	0.25 (0.555)	−0.29 (0.493)	−0.25 (0.555)
- Mucosal fibrosis		−0.02 (0.971)	−0.44 (0.275)	−0.07 (0.867)	−0.15 (0.721)
Inflammatory criteria (sum)		0.12 (0.783)	0.37 (0.367)	**0.53 **(0.176)	0.12 (0.786)
- Intraepithelial lymphocytes		0.13 (0.768)	**0.55** (0.162)	−0.19 (0.654)	0.22 (0.604)
- Lamina propria LPC		−0.26 (0.528)	0.38 (0.359)	0.11 (0.805)	0.40 (0.326)
- Lamina propria eosinophils		−0.23 (0.577)	0.03 (0.952)	0.31 (0.455)	−0.17 (0.694)
- Lamina propria neutrophils		**0.66** (0.074)	−0.25 (0.555)	**0.57** (0.139)	0.08 (0.846)
- Lamina propria ΜΦ		**0.66** (0.074)	−0.25 (0.555)	**0.57** (0.139)	0.08 (0.846)
		Colon (*n* = 9)		Colon (*n* = 9)	
Colon (composite score)		0.24 (0.527)	0.44 (0.241)	0.49 (0.179)	**0.80 (0.009; 0.009)**
Morphologic criteria (sum)		−0.32 (0.399)	0.12 (0.760)	−0.31 (0.430)	0.49 (0.184)
- Epithelial injury		−0.37 (0.329)	0.00 (1.000)	−0.19 (0.626)	0.41 (0.268)
- Goblet cell loss or hyperplasia		−0.24 (0.527)	−0.27 (0.476)	−0.13 (0.749)	0.14 (0.725)
- Crypt dilation and distortion		−0.37 (0.333)	0.05 (0.907)	−0.19 (0.626)	0.30 (0.438)
- Mucosal fibrosis and atrophy		0.06 (0.875)	0.21 (0.593)	−0.19 (0.626)	0.21 (0.593)
Inflammatory criteria (sum)		0.31 (0.427)	0.48 (0.197)	**0.60** (0.088)	**0.68 (0.046; 0.092)**
- Intraepithelial lymphocytes		**0.62** (0.077)	−0.26 (0.500)	0.32 (0.407)	−0.35 (0.361)
- Lamina propria LPC		0.07 (0.856)	**0.51** (0.157)	**0.53** (0.144)	**0.84 (0.005; 0.025)**
- Lamina propria eosinophils		**0.65** (0.058)	**0.55** (0.127)	**1.00 (<0.001; <0.005)**	**0.55** (0.127)
- Lamina propria neutrophils		−0.24 (0.527)	0.41 (0.272)	−0.13 (0.749)	0.27 (0.476)
- Lamina propria ΜΦ		–0.24 (0.527)	0.41 (0.272)	–0.13 (0.749)	0.27 (0.476)

Note: LPC: lymphocytes/plasma cells; MA: mucosal atrophy; ΜΦ: macrophages; N/A: not applicable; ^#^ lesions evaluated for fundus and antrum (combined). Cells with numbers in red indicate a statistically significant correlation (*p* < 0.05), those cells with numbers in green indicate a trend for a significant correlation (*p* < 0.1). Correlation coefficients in bold font are >0.5 or <−0.5.

## Data Availability

Data (anonymized) are available from the first or last author upon reasonable request.

## Supplementary Materials

The following supporting information can be downloaded at: https://www.mdpi.com/article/10.3390/ani12162044/s1, Figure S1: CD staining for T and B lymphocyte populations. Positive staining (brown) for CD3 (T cells; panels A&C) and CD20 (B cells; B&D) in the duodenal mucosa of a cat diagnosed with small-cell lymphoma (A&B) and a cat with CIE (C&D); Figure S2: Intestinal blood vessels containing polymorphonuclear cells. (A) S100A8/A9 immunohistochemistry (IHC), (B) S100A12+ cells within an intestinal blood vessel, (C) negative IHC control; Figure S3: Slight nonspecific background staining (S100A8/A9-IHC) of gastric parietal cells. Table S1: Patient characteristics, clinical findings, and clinicopathologic parameters in cats with chronic inflammatory enteropathy (CIE; *n* = 16), alimentary lymphoma (*n* = 8), and controls without histologic lesions (*n* = 16) included in the study; Table S2: Correlation of mucosal S100/calgranulin-positive cell counts with endoscopic lesions in cats with chronic enteropathy in this study.
