# Supplementary material for: Intestinal S100/Calgranulin Expression in Cats with Chronic Inflammatory Enteropathy and Intestinal Lymphoma
Source: Animals (Basel). 2022 Aug 11;12(16):2044. doi: 10.3390/ani12162044 (PMC9404432; doi:10.3390/ani12162044)
Supplement: Supplementary file 1 [file animals-12-02044-s001.zip › animals-1842774-supplementary.pdf]

Supplementary Materials

# Intestinal S100/Calgranulin Expression in Cats with Chronic Inflammatory Enteropathy and Intestinal Lymphoma

**Denise S. Riggers [1], Corinne Gurtner [2], Martina Protschka [3], Denny Böttcher [4], Wolf von Bomhard [5], Gottfried Alber [3], Karsten Winter [6], Joerg M. Steiner [7] and Romy M. Heilmann [1,*]**

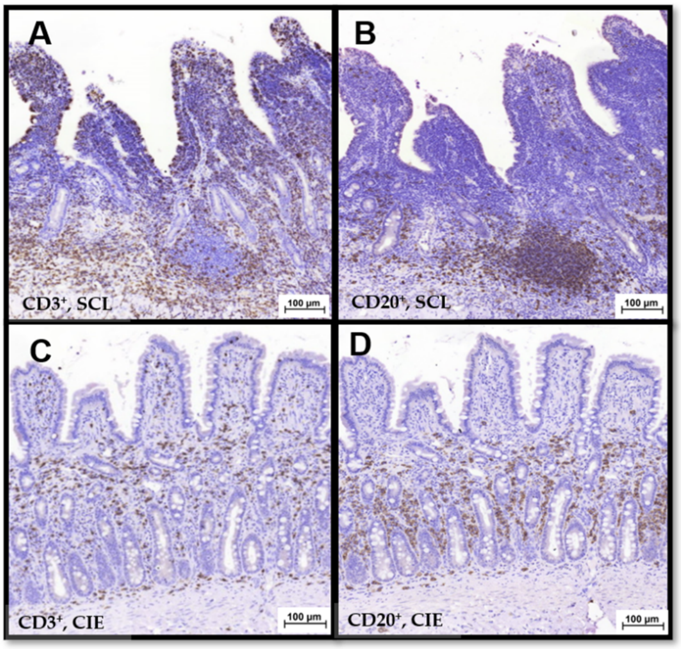


**Figure S1.** CD staining for T and B lymphocyte populations. Positive staining (brown) for CD3 (T cells; panels A&C) and CD20 (B cells; B&D) in the duodenal mucosa of a cat diagnosed with small-cell lymphoma (A&B) and a cat with CIE (C&D).

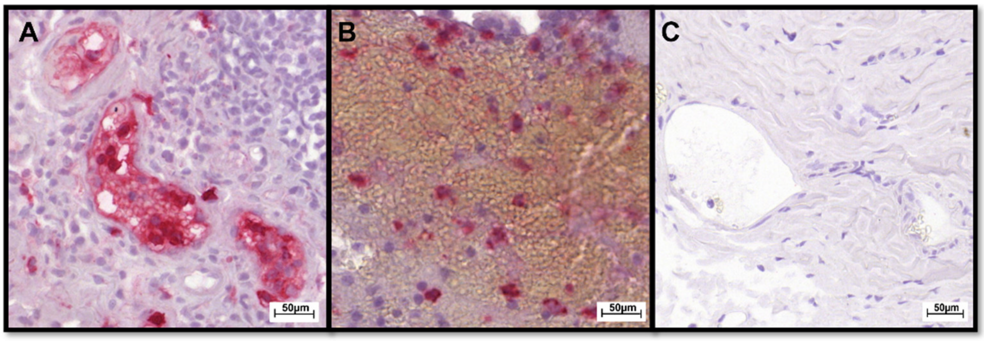


**Figure S2.** Intestinal blood vessels containing polymorphonuclear cells. (A) S100A8/A9 immunohistochemistry (IHC), (B) S100A12+ cells within an intestinal blood vessel, (C) negative IHC control.

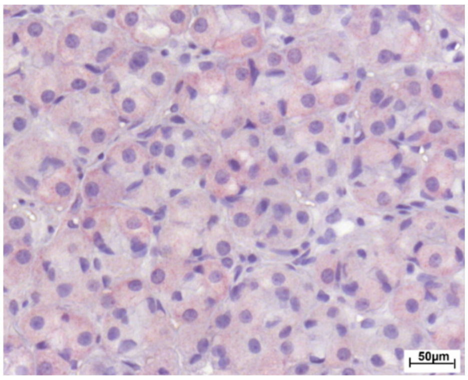


**Figure S3.** Slight nonspecific background staining (S100A8/A9-IHC) of gastric parietal cells.

**Table S1.** Patient characteristics, clinical findings, and clinicopathologic parameters in cats with chronic inflammatory enteropathy (CIE; *n* = 16), alimentary lymphoma (*n* = 8), and controls without histologic lesions (*n* = 16) included in the study.

| Patient Characteristic | CIE | Lymphoma[#] | Controls |
|---|---|---|---|
| Age in years, median (IQR) | **6.5 (2.5–12.0)**[A] | **12.0 (9.5–13.0)**[B] | 6.5 (0.5–13.5)[*,A,B] |
| Sex, male (neutered) / female (spayed) | 12 (11) / 4 (4) | 4 (4) / 4 (4) | 8 (4) / 7 (6)[†] |
| Body weight in kg, median (IQR) | 4.2 (3.0–4.8) | 3.5 (2.5–4.0) | 3.9 (2.6–4.4)[$] |
| Breed, *n* (%) | | | |
| - Domestic (European) Shorthair | 12 (75%) | 8 (100%) | 8 (67%)[‡] |
| - other breeds | 4 (25%) | 0 | 4 (33%)[‡] |
| Negative retrovirus (FeLV/FIV) status | 10 (100%)[§] | 8 (100%) | – |
| Clinical signs present in months, median (IQR) | 5.0 (2.0–12.0) | 2.5 (1.0–19.5) | – |
| Survival time in months, median (IQR) | 24.0 (7.0–45.0) | 18.0 (6.0–33.0) | – |
| Number of sites biopsied, median (IQR) | 3 (2–4) | 2 (2–3) | 2 (1–3) |
| Number of biopsies per site, median (IQR) | | | |
| - stomach[¶] | 8 (5–9) | 8 (6–10) | 2 (1–2) |
| - duodenum/prox. jejunum[**] | 7 (3–11) | 7 (2–7) | 3 (1–4) |
| - ileum[†] | 2 (1–5) | 5 (1–8) | 1 (1) |
| - colon[$$] | 6 (5–8) | 5 (5) | 1 (1) |
| | ***Clinical parameters*** | | |
| FCEAI score, median (IQR) | **7 (4–10)**[A] | **11 (9–12)**[B] | |
| - mild clinical activity (score of 0–5), *n* (%) | 6 (38%) | 1 (13%) | |
| - moderate clinical activity (score of 6–12), *n* (%) | 10 (62%) | 5 (62%) | |
| - severe clinical activity (score of 13–19), *n* (%) | 0 | 2 (25%) | |
| | | | – |
| - severity of vomiting | 1 (1–2) | 1 (1–1.5) | |
| - severity of diarrhea | 1.5 (0–2) | 1.5 (0–2) | |
| - severity of weight loss | **2 (0–2)**[A] | **3 (2–3)**[B] | |
| - severity of hyporexia | 1 (0–2) | 2 (1–2) | |
| Presence of endoscopic lesions, *n* (%) | 13 (85%) | 8 (100%) | |
| - stomach | - 8/13 (62%) | - 2/2 (100%) | |
| - duodenum | - 9/13 (69%) | - 6/6 (100%) | – |
| - ileum | - 4/6 (67%) | - 3/3 (100%) | |
| - colon | - 7/11 (64%) | - 1/1 (100%) | |
| Presence of dermatological signs, *n* (%) | 2 (13%) | 1 (13%) | – |
| | ***Clinicopathologic parameters*** | | |
| Serum cobalamin in pmol/L, median (IQR) | 257 (111–1,002) [†] | 102 (77–155) | – |
| Hypocobalaminemia (<199 pmol/L), *n* (%) | **7 (44%)**[†,A] | **7 (88%)**[B] | – |
| Serum folate in nmol/L, median (IQR) | 40.0 (23.0–48.0)[§] | 38.5 (21.1–65.4)[$] | – |
| Hypofolatemia (<25.2 nmol/L), *n* (%) | 3 (19%)[§] | 2 (25%)[$] | – |
| Hyperfolatemia (>49.0 nmol/l), *n* (%) | 2 (13%)[§] | 2 (25%)[$] | – |
| Serum total protein in g/L, median (IQR) | 69.0 (59.5–79.0) | 71.5 (66.5–75.5) | – |
| Hypoproteinemia (<59 g/L), *n* (%) | 3 (19%) | 1 (13%) | – |
| Hyperproteinemia (>87 g/L), *n* (%) | 0 | 1 (13%) | – |
| Serum albumin in g/L, median (IQR) | 28.0 (24.5–34.0) | 28.5 (24.8–32.5) | – |
| Hypoalbuminemia (<27 g/L), *n* (%) | 8 (50%) | 4 (50%) | – |
| Serum globulin in g/L, median (IQR) | 40.0 (31.3–46.3) | 40.5 (38.0–48.3) | – |

| | | | |
|---|---|---|---|
| Hyperglobulinemia (>47 g/L), *n* (%) | 3 (19%) | 2 (25%) | – |
| Serum total calcium in mmol/L, median (IQR) | 2.25 (2.18–2.49) † | 2.28 (2.23–2.45) | – |
| Total hypocalcemia (<2.2 mmol/L), *n* (%) | 7 (44%)† | 2 (25%) | – |
| Serum BUN in mmol/L, median (IQR) | 10.1 (7.5–12.0) | 10.6 (8.6–16.1) | – |
| Serum BUN increase (<5.7 mmol/L), *n* (%) | 1 (6%) | 2 (25%) | – |
| Serum phosphorus in mmol/L, median (IQR) | 1.3 (1.1–1.6) † | 1.1 (0.9–1.4) | – |
| Hypophosphatemia (<0.8 mmol/L), *n* (%) | 0† | 1 (13%) | – |
| Serum ALT activity in U/L, median (IQR) | 55 (36–64) | 63 (48–124) | – |
| Serum ALP activity in U/L, median (IQR) | 28 (17–43) † | 32 (28–44) | – |
| Serum tT4 in nmol/L, median (IQR) | 24.5 (20.0–27.7)†† | 24.5 (17.4–28.8) | – |
| Serum fPLI in µg/L, median (IQR) | 2.1 (1.1–3.5)‡‡ | 3.3 (1.8–5.9)$ | – |
| Serum fructosamine in µmol/L, median (IQR) | 203 (183–247)§§ | 213 (186–240)¶¶ | – |
| Serum fructosamine increase (>340 µmol/L, *n* (%) | 1 (9%) | 0 | |
| | ***Sonographic abnormalities*** | | |
| Increased GI wall thickness, *n* (%) | 9 (56%) | 4 (50%) | – |
| Thickened tunica muscularis layer, *n* (%) | 12 (75%) | 4 (50%) | – |
| Loss of GI wall layering, *n* (%) | 4 (25%) | 3 (38%) | – |
| Enlarged regional lymph nodes, *n* (%) | 11 (69%) | 6 (75%) | – |
| Evidence of free abdominal fluid, *n* (%) | 4 (25%) | 5 (63%) | – |

Note: FCEAI: feline chronic enteropathy activity index; GI: gastrointestinal; IQR: interquartile range; PCV: packed cell volume; WBC: white blood cell. #2 cats in this group had repeated endoscopy 5 and 29 months after initial diagnosis (due to disease recurrence or relapse) but are entered only once in this group. Parameters in bold font indicate significant differences at $p < 0.05$. *available from *n* = 14 cats; $available from *n* = 6 cats; †available from *n* = 15 cats; ‡available from *n* = 12 cats; §available from *n* = 10 cats; ¶available from *n* = 35 cats; **available from *n* = 27 cats; $$available from *n* = 19 cats; ††available from *n* = 9 cats; ‡‡available from *n* = 13 cats; §§available from *n* = 11 cats; ¶¶available from *n* = 7 cats.

**Table S2.** Correlation of mucosal S100/calgranulin-positive cell counts with endoscopic lesions in cats with chronic enteropathy in this study.

| | Segmental S100A8/A9+ cell counts | | | | Segmental S100A12+ cell counts | | | |
|---|---|---|---|---|---|---|---|---|
| | Epithelium | | Lamina propria | | Epithelium | | Lamina propria | |
| **Diagnostic imaging variable** | **Median (range)** | ***p*** | **Median (range)** | ***p*** | **Median (range)** | ***p*** | **Median (range)** | ***p*** |
| | | | | ***Endoscopy*** | | | | |
| | | | | Stomach | | | | |
| normal endoscopy | **0 (0)** | **0.028** | 0 (0–3) | 0.026 | 0 (0) | 0.059 | **0 (0–0.5)** | **0.044** |
| endoscopic lesions | **0 (0–3.5)** | | 0 (0–2.5) | | 0 (0–2) | | **0 (0–1.5)** | |
| | | | | Duodenum/proximal jejunum | | | | |
| normal endoscopy | 0.5 (0–2) | 0.125 | 4 (1–21.5) | 0.490 | **0.5 (0–3.5)** | **0.041** | 3.5 (1–10.5) | 0.873 |
| endoscopic lesions | 0 (0–0.5) | | 8 (0–13.5) | | **0 (0–0.5)** | | 4.5 (0.5–10.5) | |
| | | | | Ileum | | | | |
| normal endoscopy | 0.5 (0.5) | N/A* | 10 (10) | N/A* | 0 (0) | N/A* | 7.5 (7.5) | N/A* |
| endoscopic lesions | 0 (0–0.5) | | 3 (0.5–12) | | 0 (0–0.5) | | 2 (0.5–9.5) | |
| | | | | Colon | | | | |
| normal endoscopy | 0.5 (0–0.5) | 0.077 | 2.5 (1–3.5) | 0.151 | 0 (0) | 0.0803 | 2 (0.5–3) | 0.361 |
| endoscopic lesions | 0 (0–0.5) | | 5.5 (1.5–10.5) | | 0 (0–1) | | 5 (0.5–9) | |

*N/A: not applicable (statistical comparison not performed as data in one group only available from *n* = 1 cat). Parameters in bold font and green indicate significant differences at $p < 0.05$.
